# Effect of Marine Microalga *Chlorella pyrenoidosa* Ethanol Extract on Lipid Metabolism and Gut Microbiota Composition in High-Fat Diet-Fed Rats

**DOI:** 10.3390/md16120498

**Published:** 2018-12-09

**Authors:** Xuzhi Wan, Tiantian Li, Dan Liu, Yihan Chen, Yuanyuan Liu, Bin Liu, Huiying Zhang, Chao Zhao

**Affiliations:** 1 College of Food Science, Fujian Agriculture and Forestry University, Fuzhou 350002, China; wxz951317@163.com (X.W); litiantian_sweet@163.com (T.L.); LiuDan379@163.com (D.L.); Yihan.chen18@hotmail.com (Y.C.); liuyyai@163.com (Y.L.); 2 Fujian Province Key Laboratory for the Development of Bioactive Material from Marine Algae, Quanzhou Normal University, Quanzhou 362000, China; 3 College of Life Sciences, Fujian Agriculture and Forestry University, Fuzhou 350002, China; zhanghuiying_1988@163.com

**Keywords:** *Chlorella pyrenoidosa*, lipid metabolism, hypolipidemic, gut microbiota, AMPK signaling pathway

## Abstract

Effects of marine microalga *Chlorella* *pyrenoidosa* 55% ethanol extract (CPE55) on lipid metabolism, gut microbiota and regulation mechanism in high fat diet-fed induced hyperlipidaemia rats were investigated. Structure characterizations of major compounds in CPE55 were determined by ultra-performance liquid chromatography-quadrupole/time of flight mass spectrometry (UPLC-Q-TOF-MS/MS). The compositions of gut microbiota in rats were analyzed by high-throughput next-generation 16S rRNA gene sequencing. Oral administration with CPE55 markedly alleviated dyslipidemia through improving adverse blood lipid profile and inhibiting hepatic lipid accumulation and steatosis. CPE55 has downregulated the gene expression levels of acetyl CoA carboxylase, sterol regulatory element-binding transcription factor-1c, and 3-hydroxy-3-methyl glutaryl coenzyme A reductase and upregulated adenosine 5′-monophosphate-activated protein kinase-α. It has also improved the abundance of bacteria *Alistipes*, *Prevotella*, *Alloprevotella*, and *Ruminococcus1* and decreased the abundances of *Turicibacter* and *Lachnospira*. *Turicibacter* and *Lachnospira* were both positive correlations of metabolic phenotypes. The findings above illustrated that CPE55 might be developed as food ingredients to ameliorate lipid metabolic disorders and hyperlipidaemia.

## 1. Introduction

Lipid metabolism disorder (LMD) is a major health burden, which is associated with hyperlipidemia, dyslipidemia, cardiovascular, and other metabolic syndrome (MetS) [1,2]. Hyperlipidemia is a significant risk factor for cardiovascular and atherosclerotic diseases, which are characterized by higher levels of triglyceride (TG), total cholesterol (TC), and low-density-lipoprotein cholesterol (LDL-C) and is accompanied by lower levels of high-density-lipoprotein cholesterol (HDL-C) [3]. With considerable changes in lifestyle, the number of patients with LMD has increased dramatically in recent years. The incidence of Asian LMD is gradually approaching the level of Western countries [4]. However, many studies of LMD were focus on investigating urban and rich areas. International Diabetes Federation counted that there was one quarter of people that get LMD in the whole world [5]. Although the development of lipid-lowering drugs has made great progress, the toxic side effects of these drugs, such as liver and kidney damage, gastrointestinal reactions, and antibiotic resistance have limited its clinical application [6]. Thus, there is an urgent need to find the effective hypolipidemic compounds from natural biological ingredients to inhibit and cure LMD.

An increasing number of hypolipidemic compounds are developed from edible algae in the past few years [7]. *Chlorella pyrenoidosa*, a single-cell marine microalga, belongs to the class Chlorophyceae and it has many special biologically active ingredients. *C. pyrenoidosa* contains β-carotene, chlorophylls, polysaccharides, and polyunsaturated fatty acids, especially eicosapentenoic and docosahexenoic acids [8,9]. It is considered to be a preventive nutrient for functional and therapeutic effects in the 21st century. In recent years, *C. pyrenoidosa* or its extracts possess a variety of pharmacological effects, such as anti-tumor [10], anti-oxidant [11], antibacterial [12], anti-inflammatory [13], anti-allergic [14], and immunoregulatory [15] activities. Meanwhile, *C. pyrenoidosa* could prevent arteriosclerosis and cardiovascular diseases [16]. In addition, it also plays the hypolipidemic roles by decreasing corresponding lipid metabolic parameters and regulating cholesterol synthesis and excretion mechanism [17].

The adenosine 5′-monophosphate-activated protein kinase (AMPK) signaling pathway can regulate glycolipid metabolism [18]. Sterol regulatory element-binding transcription factor-1c (SREBP-1c) preferentially regulated genes that are involved the synthesis of fatty acid [19]. 3-Hydroxy-3-methyl glutaryl coenzyme A reductase (HMG-CoA) is considered to be the first rate-limiting enzyme in the mevalonate (MVA) pathway [20]. Acetyl CoA carboxylase (ACC) is an enzyme that limits fatty acid synthesis, which reduces β-oxidation of long-chain fatty acids [21]. Furthermore, growing evidences have demonstrated that gut microbiota is highly associated with host energy metabolism and serum lipid levels [22]. The changes of the compositions of gut microbiota can cause LMD [23]. However, pathogenic bacteria can increase gut barrier disruption and lead to inflammation, obesity-induced insulin resistance, and hyperlipidemia [24,25]. The imbalance of *Bacteroidetes* and *Firmicutes* can influence the obesity-driven disorders [26]. Some bacterial genera, such as *Ruminococcus_*1 and *Ruminococcaceae_*UCG-010, were negatively correlated with body weight and TC level, while being positively correlated with the HDL level [27]. Moreover, algae can alter the composition of microbiota and contribute microbiota to have a beneficial effect on the host [28,29]. However, the hypolipidaemic regulation mechanism of marine microalgae *Chlorella pyrenoidosa* 55% ethanol extract (CPE55) on molecular mechanism and intestinal flora in vivo has not been well reported. Therefore, in present research, the hypolipidaemic potential of CPE55 was assessed to ameliorate lipid metabolism disorder in high-fat diet rats. Furthermore, the targeted gene expression and the composition of gut microbiota were determined.

## 2. Results

### 2.1. Compound Detection Using UPLC-Q-TOF-MS/MS

UPLC analysis of CPE55 revealed 10 major components (Figure S1). These peaks were observed at different retention times from 0.91 to 13.84 min, and attempts were made to identify these components explicitly based on QTOF/MS (Figure S2). MS analysis mainly confirmed that most compounds belong to polyunsaturated fatty acids (Table S1). Partial fragment ions on *m*/*z* are consistent with previously reported data. The definite structure of each compound was identified by contrasting with those *m*/*z* spectral data reported in the documents.

### 2.2. Effect of CPE55 on Body Weight and Serum Lipids Parameters of Hyperlipidemic Rats

**Figure 1:**
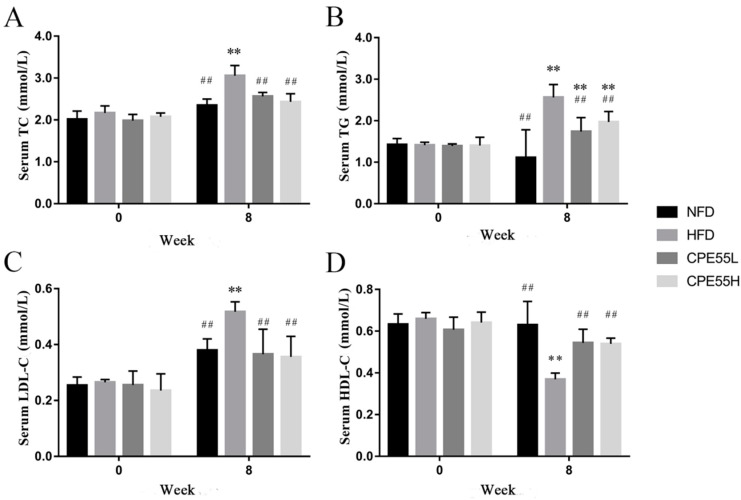
*C. pyrenoidosa* 55% ethanol extract (CPE55) prevents high-fat-diet induced hyperlipidemic rats in the eight weeks. Serum total cholesterol (TC) (**A**), serum triglyceride (TG) (**B**), serum low-density-lipoprotein cholesterol (LDL-C) (**C**), serum, high-density-lipoprotein (HDL) (**D**). Data are expressed as the mean ± SD (*n* = 8). One-way ANOVA with Tukey’s test. ** *p* < 0.01 for CPE55 versus NFD; ^##^ *p* < 0.01 for CPE55 versus high-fat diet (HFD).

At the beginning of the research, there was no significant difference in body weights of all the rats. After four weeks, the body weight of high fat diet-fed (HFD) group was obviously higher than normal diet-fed (NFD) group. Moreover, the gap of average body weight between these two groups has become larger after eight-week (Figure S3). However, the rats fed with CPE55 at a low dose obtained the weights slower than that of HFD rats. In addition, there were no significant differences in initial serum TG, TC, LDL-C, and HDL-C levels among the groups. After eight-week treatment, the serum TG, TC, and LDL-C levels of CPL55-treated groups were obviously lowered than HFD group (*p* < 0.01) (Figure 1). CPE55L has decreased serum TG, TC, and LDL-C by 19.2%, 47.5%, and 41.6%, respectively, while especially by 25.6%, 30.2%, and 45.3% in the CPE55H group. Moreover, the serum HDL-C levels of CPE55L and CPE55H were significantly increased by 35.9% and 38.9%. These results illustrated that *C. pyrenoidosa* 55% ethanol extracts could effective ameliorate serum parameters.

### 2.3. CPE55 Attenuates HFD-Induced Lipid Steatosis

**Figure 2:**
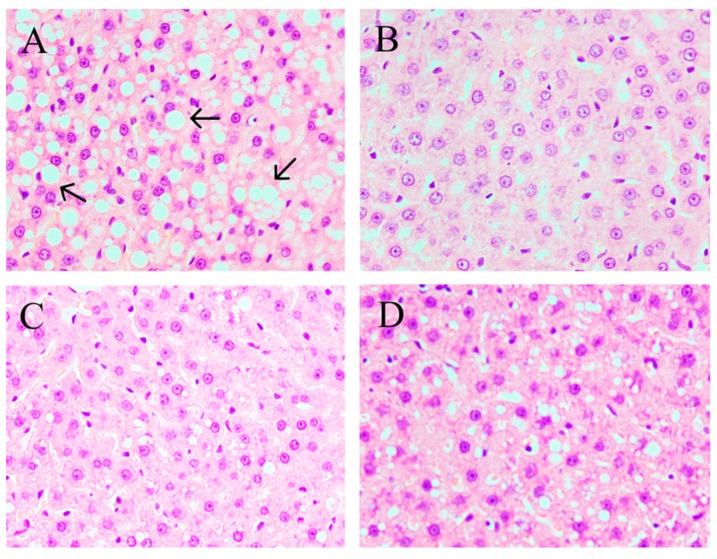
Hepatic tissues morphology analysis of CPE55 on liver tissues in the HFD (**A**), normal fat diet (NFD) (**B**), CPE55 at 150 mg/kg·day (CPE55L) (**C**), and CPE55 at 300 mg/kg·day (CPE55H) (**D**) groups at 40× magnification. Direction of the arrow indicated lipid droplets.

**Figure 3:**
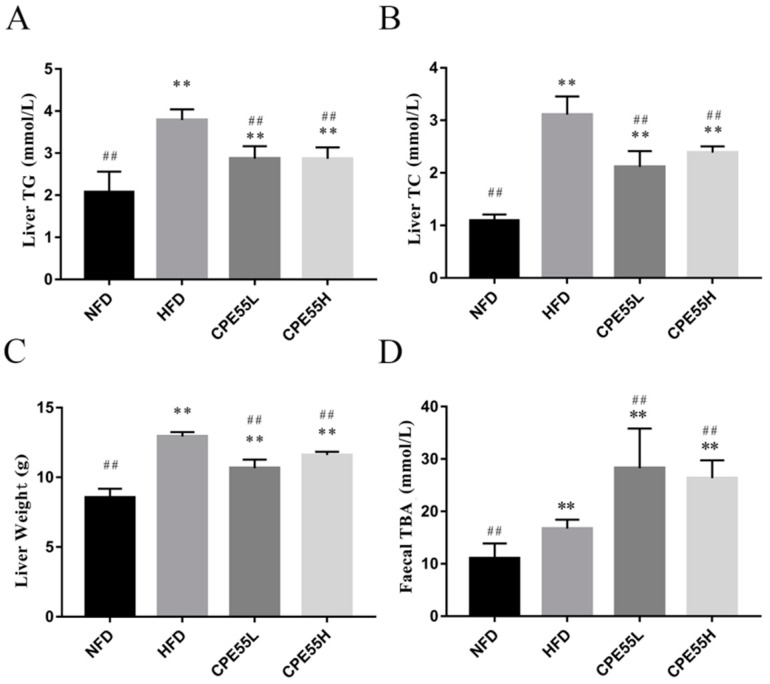
Effects of CPE55 on liver TG (**A**), TC (**B**) weights (**C**) of rats, and bile acids (TBA, **D**) content in fecal. Data are expressed as the mean ± SD (*n* = 8). One-way ANOVA with Tukey’s test. ** *p* < 0.01 for CPE55 versus NFD; ^##^ *p* < 0.01 for CPE55 versus HFD.

According to hepatic tissues morphology analysis, the sections of liver tissue in NFD group showed a normal histological architecture with integral hepatocytes, no cell rupture, and uniform distribution. In contrast, the larger lipid droplets and cell rupture were marked in the HFD group (Figure 2). The treatment with CPE55 has improved hepatic cell damage and inflammation and decreased the production of fat droplets in hyperlipemia rats (Figure 2C). Besides, the liver lipid parameters were also determined (Figure 3). The liver weights and serum TC and TG levels were significantly increased in the HFD group when compared with the NFD group, which showed that the fatty liver rat model had been successfully established. Moreover, the therapeutic effect of CPE55 was revealed by a significant decrease in lipid parameters on TC, TG, and liver weights (*p* < 0.01). The results suggested that CPE55 had displayed protective effect and decreased the fat accumulation to a certain extent.

### 2.4. Effect of CPE55 on Liver Genes and Protein Expressions

**Figure 4:**
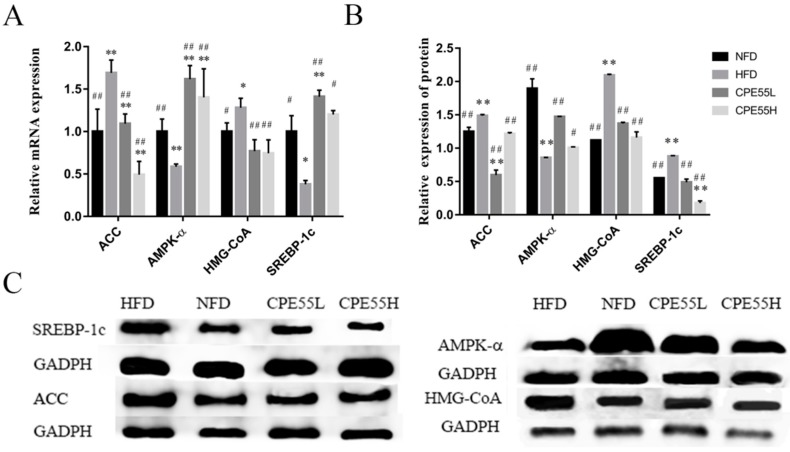
Effect of CPE55 on the mRNA and protein expressions levels in the liver. The mRNA expression (**A**) and protein expression (**B**,**C**) of Acetyl CoA carboxylase (ACC), AMPK-α, 3-Hydroxy-3-methyl glutaryl coenzyme A reductase (HMG-CoA), and Sterol regulatory element-binding transcription factor-1c (SREBP-1c) levels were determined through real-time quantitative PCR (RT-qPCR) and western blotting analysis. Data are expressed as the mean ± SD. One-way ANOVA with Tukey’s test. * *p* < 0.05 and ** *p* < 0.01 for CPE55 versus NFD; ^#^ *p* < 0.05, ^##^ *p* < 0.01 for CPE55 versus HFD.

To elucidate the molecular mechanisms of CPE55 on regulating the lipid metabolism, real-time quantitative PCR (RT-qPCR) and western blotting analysis were implemented. The current investigation revealed that there was a significant increase in the mRNA expression level of AMPK-α by CPE55 treatment when compared with the HFD group (*p* < 0.01), but was close to the situation in the NFD group. Meanwhile, CPE55 decreased the mRNA expressions of SREBP-1c, HMG-CoA, and ACC genes (*p* < 0.01) (Figure 4A). As it might be expect, CPE55 has also significantly reduced SREBP-1, ACC, and HMG-CoA (*p* < 0.01), and increased AMPK-α (*p* < 0.05) at protein levels, when compared with model group (Figure 4B,C).

### 2.5. CPE55 Regulates Caecum Microbiota Composition on Hyperlipidemic Rats

**Figure 5:**
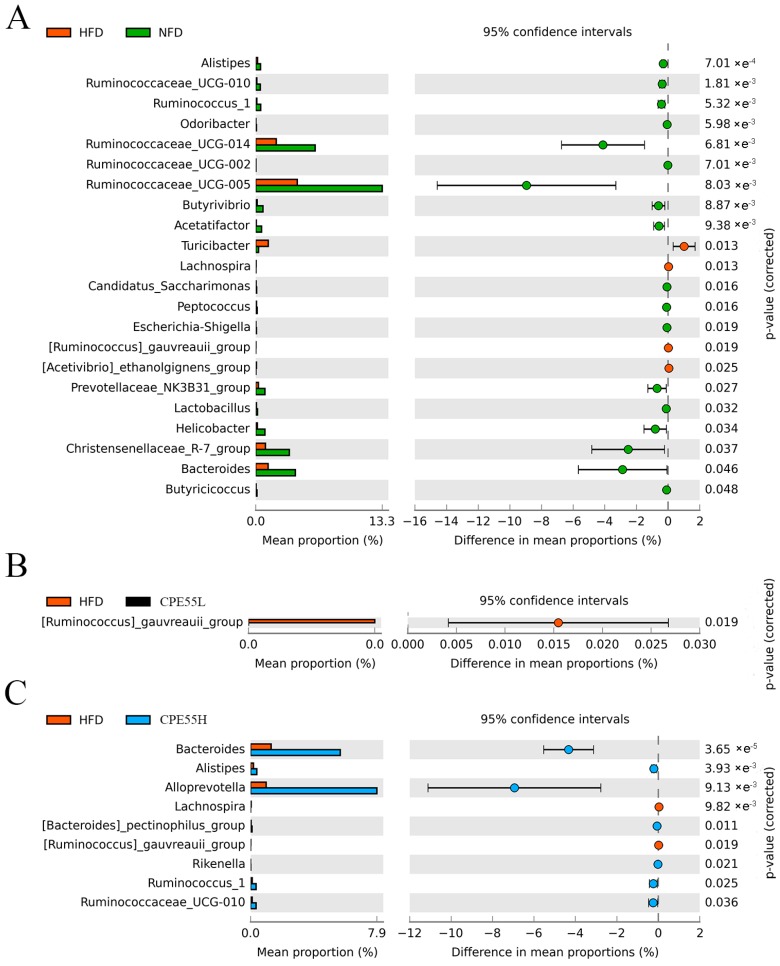
Extended error bar plot was using to determine the significant differences of average proportions of caecal bacterial taxa. T-test was used to calculate significant differences between group and the Benjamini-Hochberg method was used to correct the false discovery rate (FDR). The adjusted *p*-values were display on the right. (**A**) HFD (orange) and NFD (green); (**B**) HFD (green) and CPE55L (black); and, (**C**) HFD (orange) and CPE55H (bule).

The alternation in the composition distribution of intestinal microbiota was analyzed by using the partial least squares discriminant analysis (PLS-DA) (Figure S4). PLS-DA was generally used to construct a specific model for each class separately. The intestinal microbiota in NFD, HFD, CPE55L, and CPE55H groups were highly distinct. The HFD group mainly accumulated in the positive first principal component (PC1) and it displayed an obvious structural change when compared with the NFD group. Meanwhile, CPE55 had a certain recovery effect on the variation after feeding with HFD. Even the status of CPE55H has presented a similar trend towards the NFD group. Taxon-based analysis contained extended plots for gut microbiota profiles. The characteristic microbes of HFD group were significant increased when compared with NFD group, such as *Turicibacter*, *Lachnospira*, *Ruminococcus_gauvreauii_group*, and *Acetivibrio_ethanolgignens_group*, while the abundances of *Alistipes*, *Bacteroides*, *Ruminococcu*, and *Butyrivibrio* were lower (Figure 5). The composition of the intestinal microbiota has been truly altered in HFD-fed rats. Meanwhile, CPE55H treatment has markedly reduced the relative abundances of *Lachnospira* and *Ruminococcus_gauvreauii_group,* which were enriched in the HFD group. It also has significantly improved the relative abundances of *Alistipes*, *Bacteroides*, and *Ruminococcus*_1 and ameliorated to be similar to that of NFD group. Interestingly, *Alloprevotella* and *Ruminococcaceae_*UCG-010 were unique for CPE55H rather than NFD and HFD groups (Figure 5B). *C. pyrenoidosa* 55% ethanol extract might possess the effect on restoring the ecological imbalance of the intestinal flora and maintain its healthy composition.

### 2.6. Effect of CPE55 on Total Bile Acid in Faecal

Total bile acids (TBA) were the final products of cholesterol catabolism and closely related to absorption metabolism and the regulation of cholesterol. When compared with the NFD group, faecal TBA levels in CPE55 and HFD group were significant higher. Moreover, the TBA levels in the CPE55 group were significantly increased when compared with the HFD group (*p* < 0.01), which indicated that CPE55 had the effect on regulating the cholesterol catabolism (Figure 3D).

### 2.7. Correlation between Biological Indicators and Cecal Microbiota

**Figure 6:**
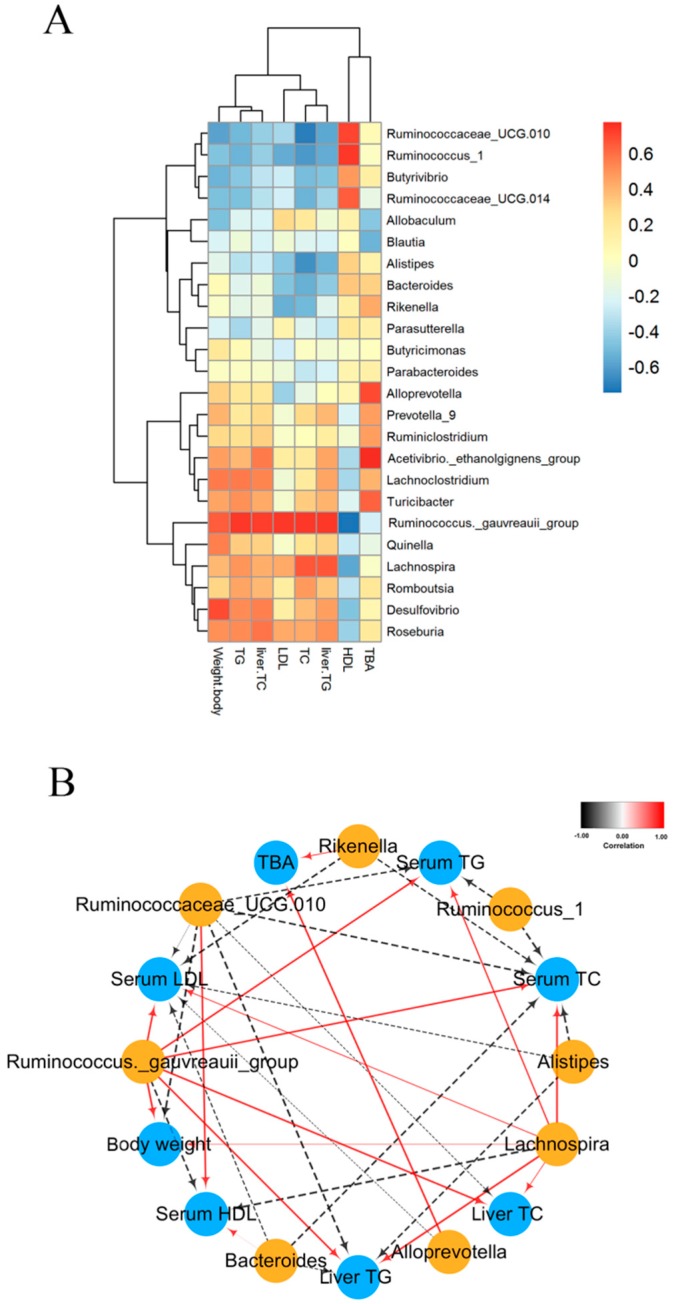
Spearman’s analysis between the microbiota and biochemical indexes at the genus level. (**A**) Heatmap shows the correlation between caecal microbiota and biochemical indexes. The depth of the color corresponds the extent of relevance between caecal microbiota and biochemical indexes. (**B**) Visualization network shows the correlation between significantly different microbiota and the biochemical indexes. Each node corresponds the intestinal flora and biochemical indexes at the genus level. The solid red line and dotted black line correspond the positive and negative correlation. Moreover, the line width demonstrates the strength of correlation. (|r| > 0.4, FDR adjusted *p* < 0.05).

The correlation between gut microbiota composition and biochemical indicators induced by CPE55 was also assessed by Spearman’s algorithm in the present research. The microbes, including *Romboutsia*, *Lachnospira*, *Roseburia*, and *Turicibacter* showed a positive relationship with abnormal parameters serum TG, TC, and LDL-C levels, and displayed a negatively correlation with the serum HDL-C levels. By contrast, *Alistipes*, *Butyrivibrio*, *Bacteroides*, *Rikenella*, and *Ruminococcus_*1 were negatively correlated with serum TG, TC, and LDL-C levels, but were positively correlated with the serum HDL-C level (Figure 6A). The network further showed that the *Alistipes*, *Bacteroides*, *Ruminococcus_*1, *Rikenella*, and *Ruminococcaceae_*UCG-010 were negative with serum lipid levels and body weights, while *Lachnospira* and *Ruminococcus_gauvreauii_group* had the positive correlations with serum parameters (Figure 6B). Interestingly, *Rikenella* and *Alloprevotella* had significantly positive correlations with TBA. These results indicated that the bacteria played the important roles in the beneficial effects of CPE55.

## 3. Discussion

A high energy diet may cause a significant raise in body weight and change in the serum and liver parameters, the abundance of gut microbiota and LMD. Prior work has revealed that *C. pyrenoidosa* administration has a wide range of pharmacological effects, including preventing fatty liver formation and having a therapeutic effect on LMD in rodent models [30]. However, the precise mechanism to ameliorate LMD has not been well investigated. This study displayed whether CPE55 possessed hypolipidemic activity and how to affect liver gene expressions and gut microbiota. Consumption of HFD obviously increased serum TG, TC, and LDL-C levels and decreased HDL-C level, which were consistent with previously reported studies [31]. CPE55 supplementation has increased serum HDL-C level and TBA, while decreased TG, TC, and LDL-C levels during eight-weeks. LDL-C and TG are considered to the essential risk factor of metabolic syndrome and cardiovascular disease. Lowering the TG levels can effectively decrease the prevalence of vascular disease [32], suggesting that CPE55 could ameliorate LMD by decreasing TG and LDL-C levels. In addition, these changes of lipid metabolic phenotypes might be associated with hepatic steatosis. In this research, the high fat diet can cause numerous lipid droplets to accumulate in the livers of rats. Histopathological analyses revealed the visibly differences of liver tissue structure and hepatic lipid accumulation in these four groups. Hepatic steatosis and lipid droplets were obviously ameliorated by CPE55 supplementation. The results illustrated that CPE55 treatment could improve the liver TG and TC levels and decrease liver weights in rats as well.

To investigate the mechanism of hypolipidemic effect of CPE55 supplementation in high fat rats, the mRNA expression levels of target genes in AMPK signaling pathway were examined, including SREBPs, ACC, AMPK-α, and HMGCR. AMPK, as an AMP-dependent protein kinase that is core to the study of hyperlipidemia and other metabolic-related diseases [18], is expressed in various metabolically related organs and activated due to the unbalanced energy metabolism of the body. The serine kinase controlled AMPK is a key molecule in the adjustment of bioenergy metabolism, especially in glycolipid metabolism [33]. It might ameliorate LMD through regulating the mRNA expression of downstream genes, such as SREBPs, ACC, and HMGCR [34]. In this investigation, *C. pyrenoidosa* supplementation regulated the expression of HMGCR, SREBP-1c, AMPK-α, and ACC, which indicating that CPE55 could ameliorate AMPK metabolic pathway in hyperlipidemia induced rats. Lipid homeostasis of cells is controlled by an intracellular cholesterol sensor located in the endoplasmic reticulum named SREBPs, which controls cholesterol synthesis by activating the related genes of SREBPs pathway. Especially, SREBP-1c as one of the main forms of SREBPs, can enhance the expression of enzyme genes that are involved in fat synthesis [19]. HMG-CoA plays an essential role in cholesterol synthesis mechanism and stimulates the synthesis of LDL receptors [20]. Thus, HMG-CoA can decrease the synthesis of cholesterol and the serum levels of LDL-C to improve hyperlipidemia disease [35]. ACC limits fatty acid synthesis by controlling the production of malonyl-CoA in the first step. Malonyl-CoA can prevent long-chain fatty acids from entering mitochondria. Indicating lower expression of ACC can cause the reduction of TG levels and increase β-oxidation. Moreover, phosphorylation of ACC inhibits the ACC activity, which decreased ACC content and reduced liver fat deposition [21]. The investigation of the gene expression levels revealed that oral administration of CPE55 improved high fat diet-fed involved hyperlipemia rats by activating the AMPK signaling pathway and inhibiting the SREBP signaling pathway, which proves the potential of CPE55 in the treatment of LMD (Figure S5).

Gut microbial community plays a crucial role in maintaining the normal physiological functions of the human body with its various functions [36]. Gut microbiota in rats was observed to elucidate the potential mechanisms for ameliorating lipid metabolism by *C. pyrenoidosa* ethanol extract. CPE55 treatment has increased the abundances of *Alistipes*, *Prevotella*, *Ruminococcus*_1, *Alloprevotella*, and *Bacteroidete*. *Prevotella* was positively correlated with serum HDL-C, whereas it was passively correlated with serum TG, TC, and LDL-C levels. The enterotype-like clusters actuated by *Prevotella* (P-type) microbiota were distinguished by a more conservative bacterial colony. P-type subjects showed higher carbohydrate digestion activity and lower biosynthesis activity of bile acid, which reflected their high abilities to resist metabolic syndrome [37]. Moreover, the recent study has shown a strong link between *Prevotella* and TBA [38]. *Prevotella* modulated the levels of lipid by altering bile acid metabolism. *Alistipes* play an important role in the improvement and treatment of intestinal diseases [39]. In addition, *Alloprevotella*, *Ruminococcus1*, and *Butyrivibrio* enriched by CPE55 were closely associated with the production of short-chain fatty acids and passively correlated with nonalcoholic fatty liver disease (NAFLD) and other chronic diseases [40]. Short-chain fatty acids can protect the intestinal mucosal barrier, inhibit inflammation, and stimulate gastrointestinal motility. Especially, butyrate is an important metabolite that is produced by bacterial fermentation of dietary fiber, which can maintain intestinal mucosal integrity and regulate intestinal flora [41]. Moreover, CPE55 has reduced the abundance of *Firmicutes* and improved the abundance of *Bacteroidetes* in caecal contents. Through the experiment, it was proved again that obesity positively correlated with the *Firmicutes* in the intestinal flora of rats [42]. Besides, the changing of body weight and serum LDL-C were associated with *Firmicutes*. The increased proportion of *Porphyromonadaceae* was connected with diabetes, NAFLD, and atherosclerosis diseases [43]. HFD enriched microbes, such as *Turicibacter,* might have a side effect on intestinal health and serum of TC or TG levels and weight gain [44].

## 4. Materials and methods

### 4.1. Preparation of C. pyrenoidosa Extract and UPLC-QTOF-MS/MS Analysis

Dried *C. pyrenoidosa* powders were obtained from King Dnarmsa *Spirulina* Co. Ltd. (Fuqing, China) and extracted using 55% ethanol at a ratio of 1:10 (*w*/*v*) and at 50 °C for 1 h. The extracts were centrifuged, concentrated, and lyophilized for further research. The components of *C. pyrenoidosa* 55% ethanol extract (CPE55) were determined on an UPLC-Q-TOF-MS/MS spectrometry analyzer (Waters, Milford, MA, USA) by C18 column (1.8 μm, 2.1 × 100 mm). According to the previous report [45], the mobile phases composing of the solvent A (0.1% formic acid (*v*/*v*) in water) and solvent B (acetonitrile). *C. pyrenoidosa* compounds were determined from 50–1200 *m*/*z*. The scan parameters were set as follows: scan time, 0.2 s, source offset, 80 V, nebulizer gas flow, 6.5 bar, capillary voltage, 2.0 kV at ESI^+^, source temperature, 120 °C, desolvation temperature, 450 °C, and nebulization gas flow, 800 L/h at 800 °C. Data acquisition was achieved using MassLynx 4.1 software (Waters, Milford, MA, USA).

### 4.2. Animals

Thirty-two male Wistar rats were purchased from Wu’s experimental animal company (Fuzhou, China). The rats were fed in a controlled environment (12 h day and night) and 60% relative humidity with a standard diet. All experimental protocols were in accordance with the guidelines of laboratory animal welfare ethics and daily animal care guidelines. The Ethics Review Committee of the College of Food Science, Fujian Agriculture and Forestry University proposed ethical approval (No. FS-2017-002). After one week of adaptation on chow diet, rats were randomly divided into four groups (*n* = 8) and fed on standard chow or high fat: normal fat diet (NFD), high-fat diet (HFD), and HFD-fed rats that were treated with CPE55 at 150 mg/kg·day (CPE55L) or at 300 mg/kg·day (CPE55H) groups. NFD group was fed on chow diet (13.5% energy from fat; Lab Diet 5001; Lab Diet, St Louis, MO, USA), while HFD and CPE55 groups were fed on HFD (67% normal diet, 20% sucrose, 10% lard, and 3% cholesterol). CPE55L and CPE55H groups were gavaged with 2 mL different concentration of CPE55 throughout eight weeks. NFD and HFD groups were instead of gavaging with 2 mL 0.9% saline. The dose used by CPE55 is based on the conversion of human dose and rats body surface area [46,47].

### 4.3. Sample Collection

Body weights were determined per week to assess their differences. The rats were fasted for 12 h and anesthetized to obtain blood specimens at first and eighth week. Blood was drawn from the heart and separated to the plasma at 3000 rpm for 15 min at 25 °C and then stored at −80 °C for use. Livers were weighed and used for histopathological analysis, partial liver was stained by haematoxylin and eosin (H&E) stain. Liver tissues were mixed with saline at a ratio 1:9 and homogenized. The mixture was centrifugated at 12,000 rpm for 10 min at 4 °C, and then the supernatant was retained for subsequent liver lipid parameters analysis.

### 4.4. Serum and Liver Biochemical Index Analysis

The levels of TG, TC, HDL-C, and LDL-C in the serum and liver tissues were determined according to the instructions of relevant assay kits (Nanjing Jiancheng Institute of Biotechnology, Nanjing, China).

### 4.5. Hepati Histopathology Analysis

The liver samples were fixed in 10% formalin solution. After paraffin embedding, the cut-off sections (5 mm) were stained with H&E and then hepatic tissues morphology were observed under the high magnification of an optical microscope with high magnification (Nikon Eclipse TE2000-U; NIKON, Tokyo, Japan) [48].

### 4.6. RT-qPCR Analysis

Using an RNA extraction kit (Takara, Kusatsu, Japan) to get total RNA, cDNA was synthesized using PrimeScript™ RT reagent Kit with gDNA Eraser (Takara, Kusatsu, Japan). RT-qPCR of control β-actin, HMG-CoA, SREBP-1c, ACC, and AMPK-α were used to monitor gene expression levels by SYBR^®^ Premix Ex Taq™ II (Takara, Kusatsu, Japan) with following the list of specific primers: ACC, F: 5′-ATGTGCCGAGGATTGATGG-3′, R: 5′-TTGGTGCTTATATTGTGGATGG-3′; β-actin, F: 5′-GGAGATTACTGCCCTGGCTCCTA-3′, R: 5′-GACTCATCGTACTCCTGCTTGCTG-3′; AMPK-α, F: 5′-TCAGGCACCCTCATATAATC-3′, R: 5′-TGACAATAGTCCACACCAGA-3′; HMG-CoA, F: 5′-TGTGGGAACGGTGACACTTA-3′, R: 5′-CTTCAAATTTTGGGCACTCA-3′; and, SREBP-1c, F: 5′-AAACCAGCCTCCCCAGAGA-3′, R: 5′-CCAGTCCCCATCCACGAAGA-3′. Using the AB7300 Real-Time PCR system to amplify the related genes (Applied Biosystems, Foster City, CA, USA). RT-qPCR procedure follows the conditions: 95 °C for 30 s, 40 cycles at 95 °C for 5 s, 60 °C for 30 s, and 72 °C for 30 s.

### 4.7. Western Blot Analysis

The hepatic tissues were homogenized in SDS lysis buffer and centrifuged at 12,000 rpm for 10 min at 4 °C. Twenty micrograms proteins were subjected to 10% SDS-PAGE and then transferred to PVDF membranes. The membranes were blocked in QuickBlock™ Blocking Buffer at 30 min. The membranes were incubated for 3.5 h at 37 °C using rabbit polyclonal antibodies against GAPDH, HMG-CoA, SREBP-1c, ACC, and AMPK-α (1:1000; Beyotime Biotechnology, Shanghai, China). The protein bands were observed using a BeyoECL Moon kit and quantified using GeneGnome XRQ chemiluminescence Imaging System.

### 4.8. Extraction of DNA from Cecal Samples and 16S rRNA Sequencing

Total metagenomic DNA was extracted from cecal contents of rats using a QIAamp-DNA stool mini kit (Qiagen, Hilden, Germany). The V3-V4 hypervariable domain of 16S rRNA gene was amplified with the special primers (F: 5′-CCTACGGRRBGCASCAGKVRVGAAT-3′ and R: 5′-GGACTACNVGGGTWTCTAATCC-3′). Sequencing was performed using a 2 × 300 paired-end configuration by IonS5^TM^XL platform. Phenotypic analysis was carried out by MiSeq control software. The initial classification analysis was conducted on Illumina’s Base Space cloud computing platform.

### 4.9. Bioinformatics Analysis

Assign high-quality sequences to samples based on barcodes. Valid sequences were denoised to investigate the diversity information of bacterial genus. Results were generated by using Usearch (Version 7.1, http://drive5.com/uparse/), with 3% disagreement. Principal components were computed to compress dimensionality into two-dimensional partial least squares discriminant analysis (PLS-DA) plots. The significant difference between two groups were displayed by an extended error bar plot using STAMP. The heatmap was produced based on the correlation between the gut microbiota and lipid metabolic parameters using RStudio software, and the visualized network of biochemical indicators and caecel microbiota correlation was generated by using Cytoscape 3.6.1.

### 4.10. Statistical Analysis

Datas for per group were expressed as mean ± SD (*n* = 8). Statistical significance was measured using one-way analysis of variance (ANOVA) with Tukey’s test. Statistical significance was expressed by a *p-*value of less than 0.05. Spearman’s rank correlation coefficient was used to assess the correlation between gut microbiota and lipid metabolic parameters.

## 5. Conclusions

It was the first research to reveal the effects of ethanol extract from *C. pyrenoidosa* on lipid metabolism in the liver by influencing relational gene expressions and reversing the shift of the serum lipid profile, and regulating gut microbiome. CPE55 could decrease the prevalence of hepatic steatosis and ameliorate hepatocyte injury according to histopathological analyses. CPE55 treatment has exerted an anti-hyperlipidemia effect by up-regulating AMPK-α and down-regulating the SREBP-1c and HMG-CoA signal pathway in liver. Its modulations on gut microbiota were also performed by selectively promoting the growth of some beneficial gut microbial community, such as *Prevotella, Butyrivibrio,* and *Alistipes*. The identification and hypolipidemic effect of ethanol extracts of *C. pyrenoidosa* has rarely been reported before this study. The potential anti-hyperlipidemia mechanism of CPE55 was characterized and a profound analysis of alternation in gut microbiota and corresponding molecular mechanisms upon CPE55 administration was conducted. As the gut bacteria cannot determine information beyond the genus levels, further work should include follow-up work to explore changes in gut flora at the species level.

## Supplementary Materials

The following are available online at http://www.mdpi.com/1660-3397/16/12/498/s1, Figure S1: Chromatographic peaks of *Chlorella pyrenoidosa* ethanol extract in UPLC, Figure S2: Representative UPLC/Q-TOF MS chromatographs of ethanol extracts of *Chlorella pyrenoidosa*, Figure S3: Effect of CPE55 on body weight of high-fat-diet rats during the experimental period, Figure S4: Principal component analysis plots of rat caecal microbiota coloured by diet, Figure S5: Summary of the mechanism of CPE55 to prevent LMD, Table S1: Characterization of probable major metabolites of CPE55 by UPLC/Q-TOF-MS/MS.
