# Supplementary material for: Effect of Marine Microalga Chlorella pyrenoidosa Ethanol Extract on Lipid Metabolism and Gut Microbiota Composition in High-Fat Diet-Fed Rats
Source: Mar Drugs. 2018 Dec 9;16(12):498. doi: 10.3390/md16120498 (PMC6315526; doi:10.3390/md16120498)
Supplement: Supplementary file 1 [file marinedrugs-16-00498-s001.pdf]

Table S1. Characterization of probable major metabolites of CPE55 by UPLC/Q-TOF-MS/MS.

No.	Rt (min)	Compound	Probable formula	Measured $[M-H]^+$ (m/z)	Representative fragmentation	References
1	0.91	Phosphatidic acid + glycerol + palmitic acid	$C_{29}H_{39}O_6$	483	**136.03**, 137.03, 152.02, **348.05**, 349.05	
2	1.44	gamma-Glutamylisol eucine	$C_{11}H_{20}N_2O_5$	260.99	144.92, 189.92, **216.05**	
3	1.67	Inosine	$C_{10}H_{12}N_4O_5$	268.07	136.03, 137.03	
4	2.72	Methyl stearate	$C_{19}H_{38}O_2$	298.07	**136.03**, 146.03, 188.04, 299.07,	
5	4.97	Gluconic acid	$C_6H_{12}O_7$	197.08	105.04, 133.07, 161.06, **179.07**, **251.00**,	
6	7.28	4,7-Dihydroxy-3-butylphthalid/iso mer methyl and-O-GluA	$C_{19}H_{24}O_{10}$	413,16	127.00, 139.00, 403.17,**412.66**	[1-8],
7	10.29	Lyso-PC (18:3)	$C_{26}H_{48}NO_7P$	518.32	**104.07**, 124.97, **184.04**	
8	10.97	Lyso-PC(18:2)	$C_{26}H_{50}NO_7P$	520.33	**104.07**, 124.97, **184.04**, 502.32	
9	12.14	Malvidin-3-O-cis-caffeoyglucoside	$C_{28}H_{31}O_{14}N_2Cl$	655.32	184.04, 449.18, 535.26, 563.26, 595.29, 596.29,	
10	13.83	Naringenin-O-glucoside-O-glucuronide	$C_{27}H_{30}O_{16}$	609	431.17, **447.20**, 519.23, 547.23, 579.26	

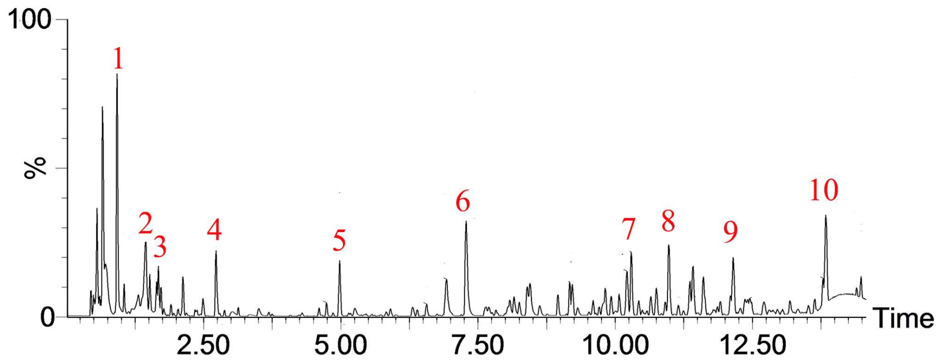

Figure S1. Chromatographic peaks of *Chlorella pyrenoidosa* ethanol extract in UPLC.

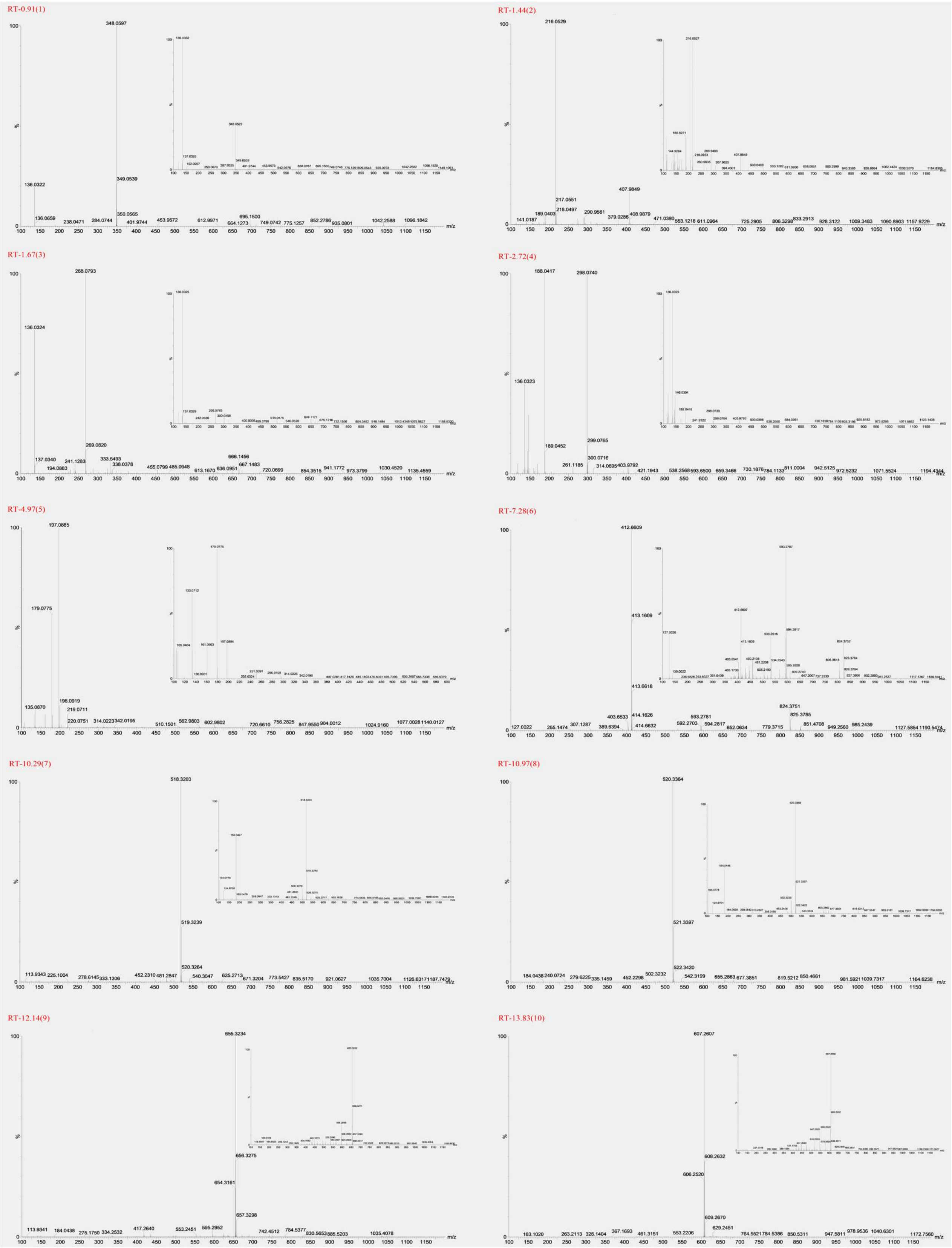

Figure S2. Representative UPLC/Q-TOF MS chromatographs of ethanol extracts of *Chlorella pyrenoidosa*.

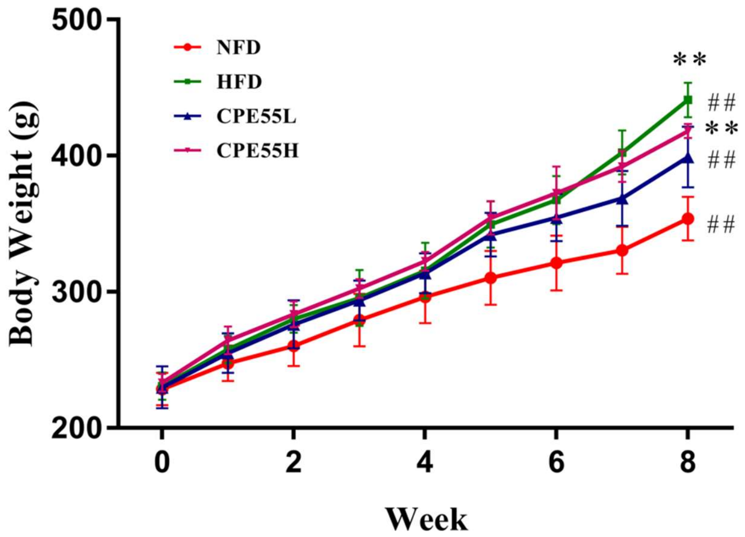

Figure. S3. Effect of CPE55 on body weight of high-fat-diet rats during the experimental period. NFD: normal fat diet; HFD: high-fat diet; CPE55L: 150 mg/(kg·day) *C. pyrenoidosa* 55% ethanol extract; CPE55H: 300 mg/(kg·day) C. pyrenoidosa 55% ethanol extract; WK: week. NFD group, rats fed NFD and gavaged with 150 mg/(kg·day) normal saline. HFD group, rats fed HFD and gavaged with 150 mg/(kg·day) normal saline. CPE55L group, rats fed HFD and gavaged with 150 mg/(kg·day) *C. pyrenoidosa* 55% ethanol extract in water. CPE55H group, rats fed HFD and gavaged with 300 mg/(kg·day) *C. pyrenoidosa* 55% ethanol extract in water. The differences were assessed by ANOVA and denoted as follows: $^{*}p$<0.05 versus the NFD group, $^{\#}p$<0.05 versus the HFD group, $^{**}p$<0.01 versus the NFD group, and $^{\#\#}p$<0.01 versus the HFD group.

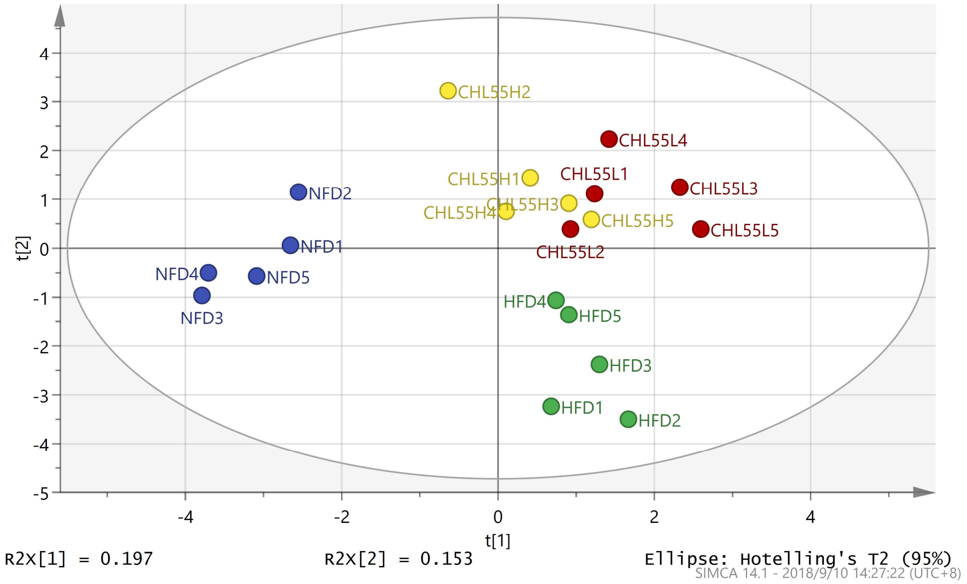

Figure S4. Principal component analysis plots of rat caecal microbiota coloured by diet. Five rats were randomly selected from each experimental group for analysis of caecal microbiota.

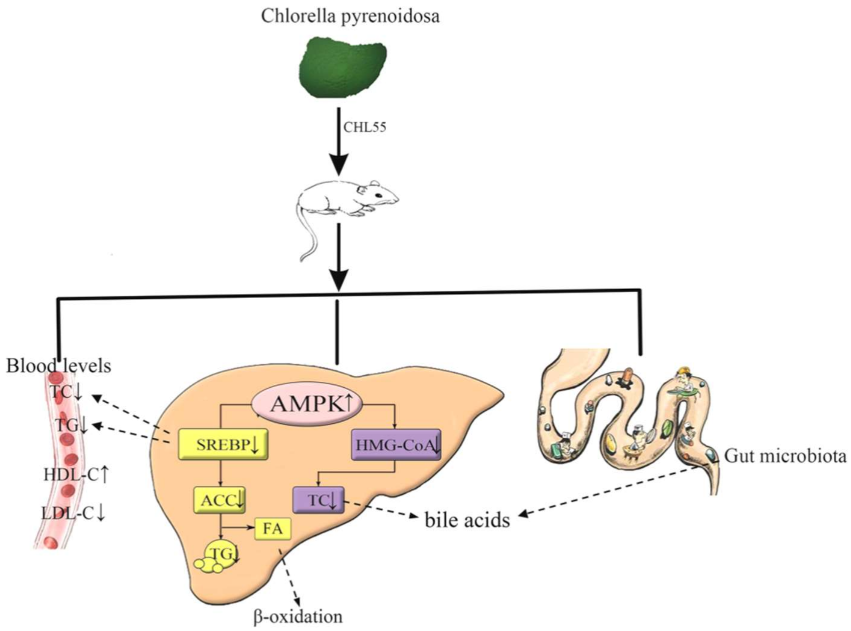

Figure S5. Summary of the mechanism of CPE55 to prevent LMD. Note: FA (fatty acids), stimulatory modification (solid arrow), and indirect modification (dotted arrow).

References

1. Herrero, M.; Vicente, M.J.; Cifuentes, A.; Ibáñez, E. Characterization by high-performance liquid chromatography/electrospray ionization quadrupole time-of-flight mass spectrometry of the lipid fraction of *Spirulina platensis* pressurized ethanol extract. *Rapid Commun. Mass Spectrom.* **2010**, *21*, 1729–1738.

2. Wu, H.; Chen, Y.; Li, Q.; Gao, Y.; Zhang, X.; Tong, J.; Zhang, Z.; Hu, J.; Wang, D.; Zeng, S.; et al. Intervention effect of Qi-Yu-San-Long Decoction on Lewis lung carcinoma in C57BL/6 mice: Insights from UPLC–QTOF/MS-based metabolic profiling. *J. Chromatogr. B* **2018**, *1102*, 22–33.

3. Zhang, L.; Ruan, Z.; Liang, J.; Du, Y.; Lu, Z.; Feng, D.; Cai, S.; Zhang, X.; Cai, W.; Hu, Z. Protective effect of propofol on ischemia-reperfusion injury detected by HPLC-MS/MS targeted metabolic profiling. *Eur. J. Pharmacol.* **2018**, *833*, 69–78.

4. Li, T. T.; Liu, Y. Y.; Wan, X. Z.; Huang, Z. R.; Liu, B.; Zhao, C. Regulatory efficacy of the polyunsaturated fatty acids from microalgae *Spirulina platensis* on lipid metabolism and gut microbiota in high-fat diet rats. *Int. J. Mol. Sci.* **2018**, *19*, 3075.

5. Mi, N.; Cheng, T.; Li, H.; Yang, P.; Mu, X.; Wang, X.; Zu, X.; Qi, X.; Guo, X.; Ye, J.; et al. Metabolite profiling of traditional Chinese medicine formula Dan Zhi Tablet: An integrated strategy based on UPLC-QTOF/MS combined with multivariate statistical analysis. *J. Pharm. Biomed. Anal.* **2019**, *164*, 70–85.

6. Suãrez-G. S.; Arola, L.; Pascual-Serrano, A.; Arola-Arnal, A.; AragonãS, G.; Bladã, C.; Suãrez, M. Development and validation of a UHPLC-ESI-MS/MS method for the simultaneous quantification of mammal lysophosphatidylcholines and lysophosphatidylethanolamines in serum. *J. Chromatogr. B Anal.* **2017**, *1055*, 86–97.

7. Deiana, M.; Loru, D.; Incani, A.; Rosa, A.; Atzeri, A.; Melis, M.; Cabboi, B.; Hollecker, L.; Pinna, M.; Argiolas, F.; et al. Wine extracts from *Sardinian* grape varieties attenuate membrane oxidative damage in Caco-2 cell monolayers. *Food Chem.* **2012**, *134*, 2105–2113.

8. Zeng, X.; Su, W.; Bai, Y.; Chen, T.; Yan, Z.; Wang, J.; Su, M.; Zheng, Y.; Peng, W.; Yao, H. Urinary metabolite profiling of flavonoids in Chinese volunteers after consumption of orange juice by UFLC-Q-TOF-MS/MS. *J. Chromatogr. B* **2017**, *1061–1062*, 79–88.
